# High prevalence and low awareness of hyperuricemia in hypertensive patients among adults aged 50–79 years in Southwest China

**DOI:** 10.1186/s12872-021-02427-2

**Published:** 2022-01-06

**Authors:** Yang Zhang, Feng-Qin Nie, Xiao-Bo Huang, Weiwei Tang, Rong Hu, Wen-Qiang Zhang, Jian-Xiong Liu, Rong-Hua Xu, Ya Liu, Dong Wei, Tzung-Dau Wang, Xu Fan

**Affiliations:** 1 Department of Cardiology, Second People’s Hospital of Chengdu, Chengdu, China; 2 Department of Endocrinology, The People’s Hospital of Wenjiang, Chengdu, China; 3 School of Health Policy and Management, Nanjing Medical University, Nanjing, Jiangsu China; 4 Division of Cardiology, The Second Affiliated Hospital of Chongqing Medical University, Chongqing, China; 5 Department of Epidemiology and Health Statistics, West China School of Public Health and West China Fourth Hospital, Sichuan University, Chengdu, Sichuan China; 6 Stroke Center, Second People’s Hospital of Chengdu, Chengdu, China; 7 Department of Geriatrics, Second People’s Hospital of Chengdu, Chengdu, China; 8 Department of Endocrinology and Metabolism, Second People’s Hospital of Chengdu, No. 10, Qingyun South Street, Chengdu, 610017 Sichuan Province China; 9 Cardiovascular Center and Division of Cardiology, Department of Internal Medicine, National Taiwan University Hospital, No. 7, Zhong-Shan South Road, Taipei City, 10002 Taiwan China; 10 Department of Public Health, CHENGDU Medical College, Sichuan, 610500 China

## Abstract

**Introduction:**

This study was aimed to assess the prevalence of hyperuricemia and its associated risk factors among hypertensive patients in Southwest China.

**Methods:**

From September 2013 to March 2014, a multistage, stratified sampling was conducted on 3505 hypertensive people aged 50–79 years who lived in urban communities within Chengdu and Chongqing, using a questionnaire and performing physical and biochemical measurements.

**Results:**

In the study population, approximately 18.2% of all hypertensive participants had hyperuricemia (638/3505), with a prevalence rate of 21.5% in men and 16.2% in women (*p* < 0.05). Multivariate logistic regression analysis showed that aging, without spouse, current drinking, preferring hotpot, hypertriglyceridemia, BMI ≥ 25 kg/ m^2^, and central obesity were all positively correlated with hyperuricemia, whereas female gender was negatively correlated with hyperuricemia. The prevalence of hyperuricemia among hypertensive patients in urban adults aged 50–79 years in southwestern China was high, while levels of awareness were extremely low.

**Discussion:**

Improved hyperuricemia health knowledge should be delivered to improve public awareness of the disease and it may need aggressive strategies aiming at the prevention and treatment of hyperuricemia. It is may necessary to encourage people to check blood uric acid levels when they first time to be diagnosed with hypertension, especially in the elderly.

## Introduction

Hypertension is the most common non-communicable disease in primary care, and a major risk factor that leads to myocardial infarction, heart failure, stroke, renal failure, and all-cause death [1–3]. The prevalence of hypertension increased from 18.8% in 2002 [4] to 29.6% in 2014 [5] in Chinese adults. Hyperuricemia is also an important independent risk factor for cardiovascular disease, stroke, and renal failure [6]. In recent years the data have shown an overall increasing prevalence of hyperuricemia among different regions [7]. In one national cross-sectional survey among Chinese adults in 2009–2010, the prevalence of hyperuricemia was 8.4% [8]. One latest meta-analysis indicated that the pooled prevalence of hyperuricemia was 13.3% in Mainland China from 2000 to 2014 [7]. Hyperuricemia plays a role in the development of hypertension [9, 10], and they often occur together. For the largest developing country-China, rapid ageing and urbanization are underway [11–13]. Rapid city development in Chongqing and Chengdu, their GDP grow up to 1774.1 billion and 1217 billion in 2016, compared to 390.7 billion and 275 billion in 2006 respectively, according to the data revealed in 2106 from the national bureau of statistics. These are causing the prevalence of hypertension and hyperuricemia increasing at an alarming rate in China [7, 8, 14, 15]. Contrary to the rapid increase in prevalence, the awareness of hypertension and hyperuricemia remained low. Hyperuricemia is often asymptomatic and less than one in ten patients with hyperuricemia presents with gout [7]. Most of hypertensive patients are asymptomatic. A study investigated urban adults in southwestern China in 2013–2014 showed that the prevalence of hypertension was 38.4%, of which only 47.9% were aware of their hypertension, 40.1% were undergoing antihypertensive treatment, and just 10.3% achieved BP control [16]. Both hypertension and hyperuricemia are independent risk factors for cardiovascular diseases, and when combined there was an escalation of risk. China is the world's largest developing country, with a vast territory and many different ethnic groups, the economic development varies greatly among different regions. However, epidemiological investigations of the prevalence of hyperuricemia in hypertensive patients in the southwestern region are lacking. In this study, we aimed to assess the prevalence of hyperuricemia among hypertensive patients, aged 50–79 years, in Southwest China and its associated risk factors.

## Materials and methods

### Study population

From September 2013 to March 2014, a multistage, stratified sampling was conducted on 3505 people aged 50–79 years who lived in urban communities within Chengdu and Chongqing, using a questionnaire and performing physical and biochemical measurements. During the first phase of this study, the Yubei and Jiangbei districts were randomly selected for Chongqing, and the Jinjiang, Longquan and Chenghua districts were randomly selected from the urban area of Chengdu. During the second phase, a random subdistrict was selected from each major district, and during the third stage, one community was randomly selected from each subdistrict, resulting in a sample consisting of five random communities.

### Inclusions and exclusions

Residents aged 50–79 years who had lived in the selected communities for more than five years and whose SBP ≥ 140 mmHg and/or DBP ≥ 90 mmHg, and/or being diagnosed with hypertension and currently under antihypertensive drug treatment, were included in the study. People with a history of mental illness, malignancies, renal failure requiring dialysis, or who refused to participate in this inquiry were excluded. From September 2013 to March 2014, 3505 patients were included in the final analysis.

### Data collection

More than 30 investigators were trained for data collection. All subjects filled out the same onsite questionnaire, according to the cardiovascular survey methods set out by the World Health Organization, which included demographic characteristics; lifestyle risk factors; personal and family histories; height, weight, WC, and blood pressure measurements [17]. The questionnaire also included the level of awareness of hyperuricemia; and type of treatment. BMI was calculated as weight (kg) divided by height (meters) squared, and when measuring height and weight, subjects were required to be barefoot and to be wearing only lightweight clothing. Investigators measured the minimum circumference between the inferior margin of the ribcage and the crest of the iliac to obtain WC measurements [18]. Patients should be seated comfortably in a quiet environment for 15 min before beginning BP measurements, subjects were told not to drink coffee, tea, or alcohol and to refrain from smoking or exercising. Two BP measurements were made on the right arm with participants in a seated position by using an automated BP monitoring device (Omron), 10 min apart, and additional measurements only if the two readings differ by > 10 mmHg. BP is recorded as the average of the two BP readings.

### Blood sample collection and laboratory tests

Venous blood was drawn after 12 h of fasting. Blood glucose, lipids, and uric acid (UA) levels were assessed in all blood samples. Patients were tested using the oral glucose tolerance test (OGTT), wherein 75 g of glucose was dissolved in 300 ml of warm water and was administered orally within five minutes, and venous blood was drawn two hours later. The total cholesterol (TC), triglycerides (TG), and blood glucose levels were detected by enzymatic methods. High-density lipoprotein cholesterol (HDL-C), low-density lipoprotein cholesterol (LDL-C) levels were measured using a homogeneous method. Serum UA was measured by the phosphotungstic acid method.

### Diagnostic standards

According to the recommendations from 2018 European Society of Cardiology (ESC) and the European Society of Hypertension (ESH) Guidelines, high blood pressure was defined as an SBP ≥ 140 mm Hg and/or a DBP ≥ 90 mm Hg and/or a diagnosis of hypertension currently treated by antihypertensive drugs [1]. Hyperuricemia was defined as serum level of uric acid > 420 μmol/L (7.0 mg/dL) for men and > 360 μmol/L (6.0 mg/dL) for women [19, 20]. DM was defined as a fasting plasma glucose (FPG) level ≥ 7.0 mmol/L, 2-h postprandial glucose (2-hPG) level ≥ 11.1 mmol/L, or a previous clinical diagnosis [21]. According to the criteria of the NCEP Adult Treatment Panel III report, hypertriglyceridemia was defined as a TG level ≥ 1.7 mmol/L, high LDL-cholesterolemia was defined as a LDL-C level ≥ 3.4 mmol/L, hypercholesterolemia was defined as a TC level ≥ 5.2 mmol/L [22]. Overweight was defined as a BMI of 25.0–29.9 kg/m^2^, and obesity was defined as a BMI of 30.0 kg/m^2^ or more [23]. Central obesity was defined as a WC of 90 cm or more in men and of 80 cm or more in women [24]. A history of smoking was defined as smoking at least once per day for more than a year, and currently having smoked or quit smoking for less than 3 years. A history of drinking was defined as drinking at least once a week over a year, and currently having drunk or quit drinking for less than 3 years. The family history of hypertension was defined as immediate family members having hypertension. The family history of DM was defined as immediate family members having DM. Physical exercise was defined as having at least one exercise session per week.

### Statistical analysis

EpiData 3.02 database software was used to record data from the questionnaires. Data input was completed by two researchers, who also performed data checking and correction, and data processing and analysis were carried out using the SAS 9.3 software (Institute Inc. SAS, Cary, NC, USA). Qualitative data were compared using Chi-square test. Quantitative data were compared using the t-test. The Chi-square linear trend test was used to detect the trend in the prevalence of hypertension with hyperuricemia in individuals in association with their age and BMI. Logistic regression was used to explore the potential risk factors for hypertension with hyperuricemia. A *P* < 0.05 was considered as significant.

## Results

The basic characteristics of the study population are shown in Table 1. In this study, 3505 hypertensive patients aged 50–79 years in Southwest were included, among whom 1338 were men and 2167 were women, with a mean age of 62.8 ± 7.6 years, and the mean serum uric acid (UA) were 309.3 ± 84.4 μmol/L. Compared to women, men had higher values of UA, age, WC, and DBP, had higher rates of drinking and smoking, and had higher personal monthly incomes and higher education levels (all *p* < 0.05). However, women had higher BMI, TC, TG, HDL-C, LDL-C, FPG, 2-hPG (all *p* < 0.05). There were no differences in SBP, heart rate, physical exercise and family history of hypertension (all *p* > 0.05).

**Table 1:** Baseline characteristics of the hypertensive population

Groups	Overall (n = 3505)	Male (n = 1338)	Female (n = 2167)	*P* values
Age, mean (SD)	62.8 (7.6)	63.7 (7.4)	62.3 (7.8)	0.000
Current smoking (%)	21.1	49.6	3.5	0.000
Current drinking (%)	17.0	37.9	4.0	0.000
Education lever high school or above (%)	17.7	29.8	10.2	0.000
Income 2000 Yuan/month or above (%)	18.6	24.4	14.9	0.000
Physical exercise (%)	62.8	64.8	61.6	0.06
Family history of hypertension (%)	24.1	25.6	23.1	0.084
Systolic pressure, mmHg, mean (SD)	170 (21.3)	171.3 (21.7)	169.5 (21.1)	0.102
Diastolic pressure/mmHg, mean (SD)	97.3 (18.6)	100.3 (21.9)	96 (16.7)	0.000
Heart rate/min, mean (SD)	82.1 (31.4)	81.8 (34.7)	82.3 (29.2)	0.639
TC, mmol/L, mean (SD)	4.7 (0.9)	4.6 (0.9)	4.9 (0.9)	0.000
HDL-C, mmol/L, mean (SD)	1.4 (0.3)	1.3 (0.3)	1.4 (0.3)	0.000
LDL-C, mmol/L, mean (SD)	2.6 (0.8)	2.5 (0.8)	2.7 (0.8)	0.000
TG, mmol/L, mean (SD)	1.8 (1.4)	1.7 (1.3)	1.9 (1.5)	0.000
FPG, mmol/l, mean (SD)	6 (2)	5.9 (1.9)	6.1 (2)	0.022
2hPG, mmol/L, mean (SD)	9 (4.1)	8.7 (3.8)	9.2 (4.2)	0.001
Uric acid, mmol/L, mean (SD)	309.3 (84.4)	352.4 (81.4)	283.2 (75)	0.000
Waist circumference, cm, mean (SD)	85.9 (27.8)	87.8 (43.1)	84.7 (9.9)	0.009
BMI, kg/m^2^, mean (SD)	25.1 (8.5)	24.7 (7.5)	25.3 (8.6)	0.022

**Fig. 1:**
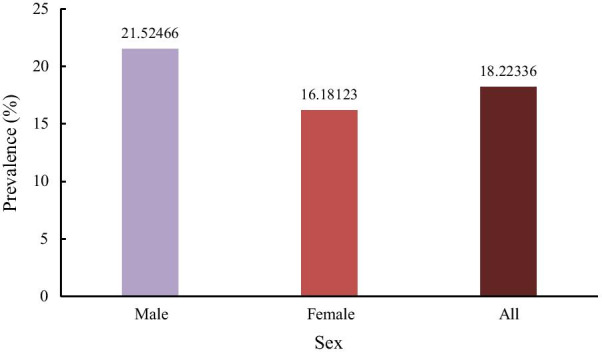
Sex-specific prevalence of hyperuricemia in hypertensive patients among adults aged 50–79 years in Southwest China

**Fig. 2:**
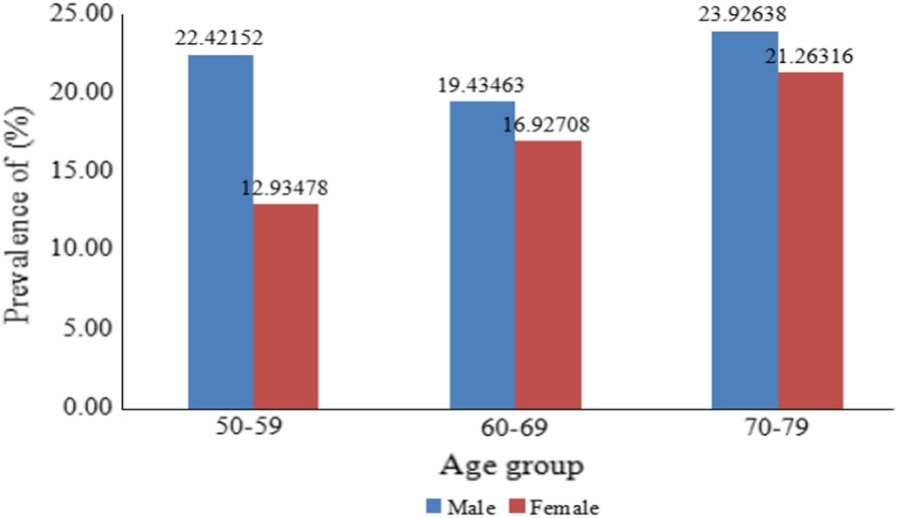
Age-specific prevalence of hyperuricemia in hypertensive patients among adults aged 50–79 years in Southwest China

**Fig. 3:**
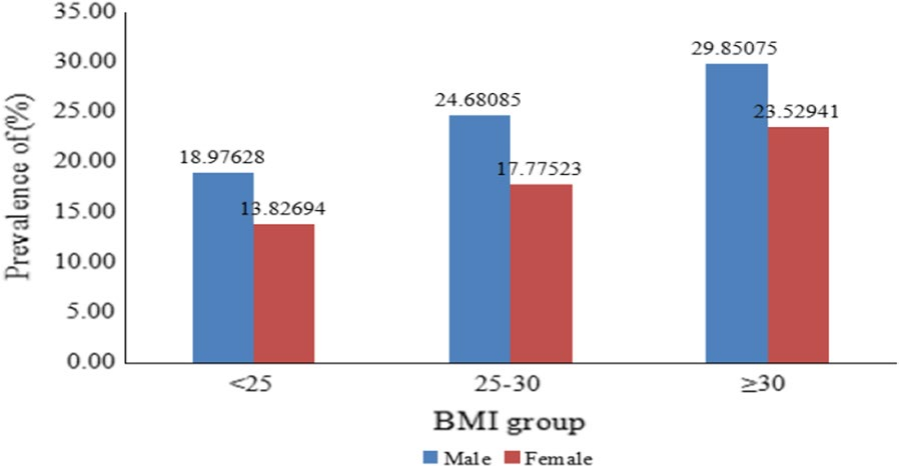
BMI-specific prevalence of hyperuricemia in hypertensive patients among adults aged 50–79 years in Southwest China

The characteristics of the prevalence of hyperuricemia among hypertensive patients are shown in Figs. 1, 2 and 3. As shown in Fig. 1, approximately 18.2% of all participants had hyperuricemia (638/3505), with a prevalence rate of 21.5% in men and 16.2% in women (*p* < 0.05). As shown in Fig. 2, the prevalence of hyperuricemia increased significant with increasing age in women (*p* for trend < 0.05), but not in men. For women in the age ranges of 50–59, 60–69, 70–79, the prevalence of hyperuricemia in hypertensive patients were 12.9%, 16.9% and 21.3% (*p* for trend < 0.05), respectively. As shown in Fig. 3, the prevalence of hyperuricemia increased with BMI in both sexes (*p* for trend < 0.05). In the BMI ranges of < 25, 25–29.9, ≥ 30 kg/m^2^, the prevalence of hyperuricemia in hypertensive patients were 19.9%, 24.7%, 29.9% in men, and 13.8%, 17.8%, 23.5% in women, respectively (*p* < 0.05).

The awareness of hyperuricemia in different groups are shown in Table 2. Among the 638 (18.2%) hyperuricemia participants, 138 (3.9%) patients were previously diagnosed, and 500 (14.3%) were newly diagnosed. This means that 78.4% (500/638) of hyperuricemia patients didn't aware of their illness. The unawareness rate was higher among women compared to men (81.4% versus 74.7%, *p* < 0.05). The unawareness rate was 84.5% in 50–59 years subgroup, then the rate decreased. In 60–69 years subgroup the unawareness rate was 74.6%, and in 70–79 years subgroup it was 76.0%.

**Table 2:** Hyperuricemia detection in hypertensive populations

Parameter	Previously diagnosed	Newly diagnosed	Proportion of newly diagnosed*
N (%)	138 (3.9)	500 (14.3)	78.4
Gender			
Male	73 (5.5)	215 (16.1)	74.7
Female	65 (3.0)	285 (13.2)	81.4
Age group			
50–59	34 (2.5)	185 (13.5)	84.5
60 ~ 69	61 (4.6)	179 (13.4)	74.6
70–79	43 (5.4)	136 (17.0)	76.0

*Linear trend χ^2^ = 4.640; *P* = 0.03

A multivariate logistic regression analysis was performed to identify significant determinants of hyperuricemia among hypertensive population, and the results are shown in Table 3. Multivariate logistic regression analysis showed that aging, without spouse, current drinking, like hotpot, hypertriglyceridemia, BMI ≥ 25, and central obesity were all positively correlated with hyperuricemia, whereas female was negatively correlated with hyperuricemia.

**Table 3:** Logistics regression for hyperuricemia among the hypertensive populations

Variable	Odds ratios (95% CI)	*P* value
Gender (male)		
Female	0.756 (0.602,0.949)	0.016
Age group (50–59)		
60–69	1.122 (0.911,1.383)	0.278
70–79	1.501 (1.185,1.901)	0.001
Marriage		
Without spouse	1.789 (1.383,2.316)	0.000
Drinking (no)		
Yes	1.342 (1.046,1.721)	0.021
Preferring hotpot (no)		
Yes	1.375 (1.099,1.721)	0.005
Hypertriglyceridemia (no)		
Yes	1.732 (1.409,2.129)	0.000
BMI ≥ 25 (no)		
Yes	1.243 (1.022,1.511)	0.029
Center obesity (no)		
Yes	1.481 (1.162,1.888)	0.002

## Discussion

This study assessed the prevalence of and factors related to hyperuricemia among hypertensive population aged 50–79 years, who lived in urban Chengdu and Chongqing, from September 2013 to March 2014. Overall, the prevalence of hyperuricemia was 18.2% among hypertensive population. In a national cross-sectional survey among Chinese adults in 2009–2010, the prevalence of hyperuricemia was 8.4% [8], and a latest meta-analysis indicated that the pooled prevalence of hyperuricemia was 13.3% in Mainland China from 2000 to 2014 [7]. The prevalence of hyperuricemia was lower than that in our study, which may be caused by factors like subjects included in this study were urban residents with hypertension and had higher mean age. Both hyperuricemia and hypertension have similar risk factors, such as age, obesity, hypertriglyceridemia and so on [8, 16]. Hence, hypertensive patients may be more likely to have hyperuricemia. Besides, as hyperuricemia was a positive risk factor for the development of hypertension [9, 10], perhaps the prevalence of hyperuricemia was higher in hypertensive patients than in the general population. Among the 638 (18.2%) hyperuricemia participants, only 138 (3.9%) patients were previously diagnosed, and 500 (14.3%) were newly diagnosed. That means 78.4% (500/638) of hyperuricemia patients had not been diagnosed and were not aware of the disease. The 78.4% unawareness rate means extremely low treatment and control rates. It is may necessary to encourage people to check blood uric acid levels when they first time to be diagnosed with hypertension, especially in the elderly.

In accordance with previous studies [5, 7], men had higher prevalence of hyperuricemia than women (21.5% vs 16.2%). Men had much higher rates of current drinking, which had already been identified as a risk factor for hyperuricemia [25, 26]. In our study, the odd ratio of current drinking was 1.34 (*P* < 0.05). In our study, female was a protective factor of hyperuricemia, this may be explained by the protective effect of estrogen [26]. In accordance with previous studies, age was confirmed as an independent risk factor for hyperuricemia in our study. However, the effect of advanced age on hyperuricemia was different between sexes. The prevalence of hyperuricemia increased significant with increasing age (*p* < 0.05) in women, but it was not observed in men. This disparity may be largely related to the loss of the uricosuric action of estrogen following menopause [26, 27]. The disparity of hyperuricemia prevalence between sexes seems to narrow with advanced age. But even so, hyperuricemia was still a male-dominant disease as indicated in our study.

Many studies have reported that hypertriglyceridemia, BMI ≥ 25, central obesity were independently risk factors for hyperuricemia [25, 26, 28–31]. In this study, multivariate logistic regression results have further confirmed the association between these metabolic abnormalities and hyperuricemia.

Hotpot is one of the representative cuisines in Chengdu and Chongqing, which is one kind of purine-rich diet, that may account for why it was an independent risk factor for hyperuricemia. The data disclosed that people without spouse was a risk factor for hyperuricemia in hypertensive patients. In geriatric patients with chronic illnesses, many studies stated that people with spouse could improve their medication compliance and quality of life [32–34]. These phenomena may explain that most of the elderly population who have a spouse eat more healthily and exercise more regularly.

Rapidly increasing prevalence in hyperuricemia, with an extremely low awareness and treatment rates, might lead to high incidences of renal failure, stroke and other cardiovascular diseases. It is may necessary to encourage people to check blood uric acid levels when they first time to be diagnosed with hypertension, especially in the elderly.

Several limitations remain. First, this is a cross-sectional study, the results cannot be used to establish a conclusive cause-and-effect relationship between risk factors and hyperuricemia in hypertensive patients. Second, the study was conducted in urban areas of Chongqing and Chengdu; hence, the results may not be representative of the prevalence of hyperuricemia in hypertensive patients among rural residents in southwestern China.

## Conclusion

The high prevalence of hyperuricemia among hypertensive patients in urban adults aged 50–79 years in southwestern China, but low levels of awareness. Strengthen the public's understanding of the harm of high uric acid and it is may necessary encourage people to check blood uric acid levels when they first time to be diagnosed hypertension, especially in the elderly. To prevention hyperuricemia, life style should be changed, such as limiting intake purine-rich diet, taking regular physical exercise, limiting alcohol and weight loss.

## Data Availability

The datasets generated and/or analysed during the current study are not publicly available due to the general data accuracy control, but are available from the corresponding author on reasonable request.
